# Multimodality imaging-guided transcatheter closure of a tortuous LCX–CS fistula: a case report

**DOI:** 10.1093/ehjcr/ytaf498

**Published:** 2025-10-04

**Authors:** Ryusuke Sekii, Shingo Kato, Kazuki Fukui, Daisuke Utsunomiya, Kiyoshi Hibi

**Affiliations:** Department of Cardiology, Yokohama City University, Fukuura 3-9-9, Kanazawa Ward, Yokohama 236-0004, Japan; Department of Diagnostic Radiology, Yokohama City University Graduate School of Medicine, 3-9, Fukuura, Kanazawa-ku, Yokohama, Kanagawa 236-0004, Japan; Department of Cardiology, Kanagawa Cardiovascular and Respiratory Center, Fukuura 3-9-9, Kanazawa Ward, Yokohama 236-0004, Japan; Department of Diagnostic Radiology, Yokohama City University Graduate School of Medicine, 3-9, Fukuura, Kanazawa-ku, Yokohama, Kanagawa 236-0004, Japan; Department of Cardiology, Yokohama City University, Fukuura 3-9-9, Kanazawa Ward, Yokohama 236-0004, Japan

**Keywords:** Coronary artery fistula, Congenital heart disease, Cardiac computed tomography, Cardiac magnetic resonance imaging, Catheter intervention, Triple coaxial system, Case report

## Abstract

**Background:**

Coronary artery fistula (CAF) is a rare congenital anomaly often detected incidentally. When large, it may cause significant left-to-right shunting, requiring closure in symptomatic cases or those with a substantial shunt volume. While previously treated surgically, transcatheter fistula closure is recognized as an effective and less invasive approach. We report a case of a large, tortuous CAF between the left circumflex artery (LCX) and the coronary sinus (CS) successfully treated with transcatheter coil embolization guided by multimodality imaging.

**Case summary:**

A 44-year-old woman presented with exertional dyspnoea and a continuous murmur. Contrast-enhanced computed tomography (CT) revealed a markedly dilated and tortuous CAF arising from the LCX and draining into the CS. Cardiac magnetic resonance imaging (MRI) demonstrated a Qp/Qs ratio of 1.50, and myocardial perfusion scintigraphy showed no ischaemia. Pre-procedural CT revealed a narrowing neck in the distal segment and severe tortuosity of the fistula. Stable access was achieved using a triple coaxial catheter system, which enabled successful coil delivery to the targeted site via an arterial approach. The procedure was completed without complication in a single session. At 6-month follow-up, cardiac MRI showed a reduced Qp/Qs ratio of 1.17, and contrast-enhanced CT confirmed occlusion of the target vessel.

**Discussion:**

Transcatheter coil embolization is a safe and effective treatment option, especially for tortuous lesions. In this case, multimodality imaging enabled accurate anatomical and functional assessment and procedural planning. The use of a triple coaxial catheter system contributed to stable access and successful coil delivery in this anatomically challenging lesion.

Learning points

- Multimodality imaging is essential for accurate anatomical and physiological assessment and procedural planning in complex coronary arteriovenous fistulas.
- Transcatheter coil embolization using a triple coaxial catheter system can be performed safely and effectively in selected patients with tortuous and complex coronary fistulas.

## Introduction

Coronary artery fistulas (CAFs) are rare congenital anomalies characterized by an abnormal communication between a coronary artery and a cardiac chamber or adjacent vessel.^1^ While most cases remain asymptomatic and are discovered incidentally, large fistulas can result in significant left-to-right shunting or myocardial steal. The decision to close a CAF depends on several factors, including symptoms, shunt volume, and the risk of long-term complications. A Qp/Qs ratio greater than 1.5 is classically considered an indication of fistula closure, even in asymptomatic patients.^2^

Transcatheter fistula occlusion has emerged as a less invasive and effective alternative to surgical ligation for suitable CAFs. Coil embolization is a particularly well-suited treatment for cases involving tortuous anatomy.^3^ Comprehensive pre-procedural assessment using multimodality imaging, including computed tomography (CT), cardiac magnetic resonance imaging (MRI), and coronary angiography, is critical in case selection and procedural planning.

We report a case of a large left circumflex artery (LCX) to coronary sinus (CS) fistula, incidentally discovered in a patient presenting with exertional dyspnoea, and associated with persistent left superior vena cava (PLSVC). The lesion was successfully closed via transcatheter coil embolization using a triple coaxial catheter system, following comprehensive anatomical assessment and procedural planning.

## Summary figure

**Figure:**
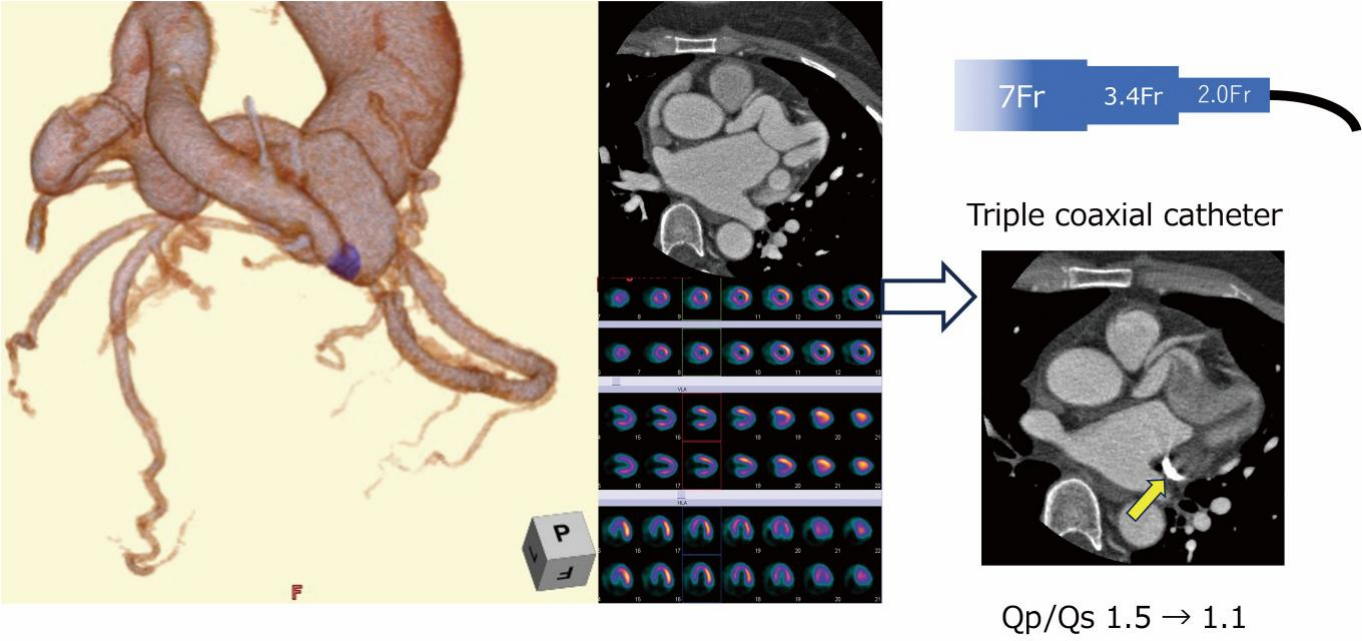


## Case presentation

**Figure 1:**
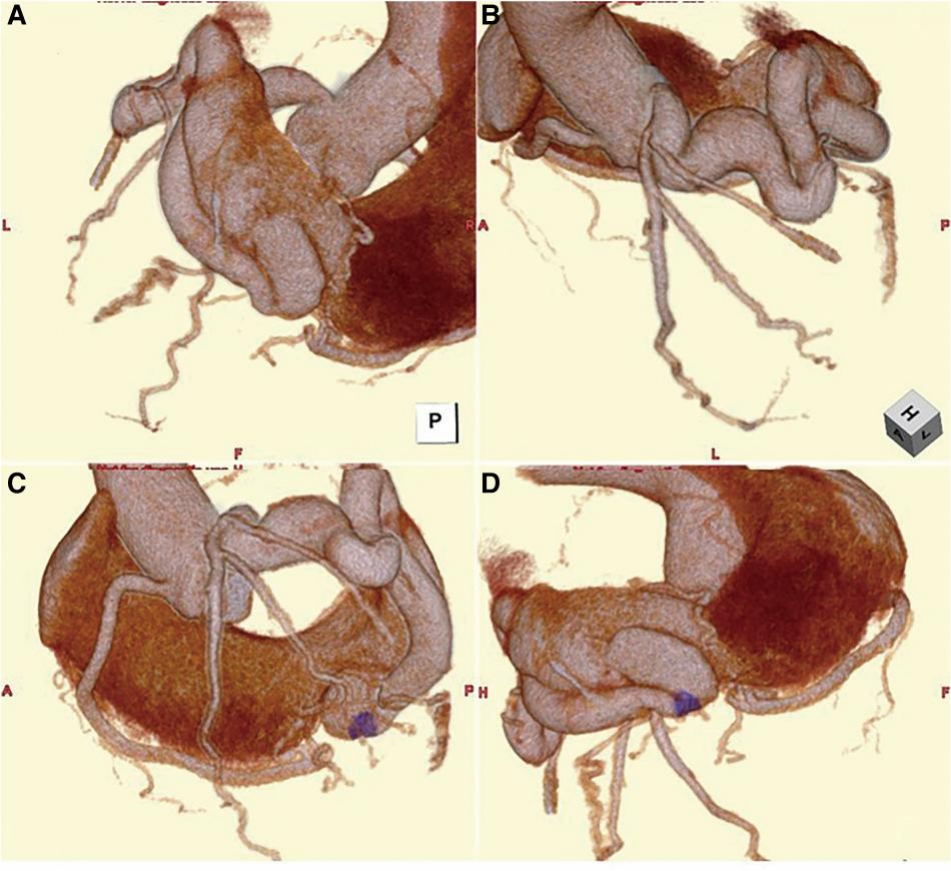
Pre-procedural contrast-enhanced computed tomography with 3D reconstruction. (*A*) Posterior view. (*B*) Left anterior cranial view. (*C*) Left anterior view. (*D*) Right posterior caudal view. Reconstructed computed tomography images reveal a markedly dilated and tortuous coronary artery fistula originating from the left circumflex artery and draining into the coronary sinus. The fistulous vessel demonstrates considerable length and multiple curves. A discrete neck segment (highlighted in purple) was identified in the distal portion and selected as the target site for transcatheter coil embolization based on pre-procedural planning.

**Figure 2:**
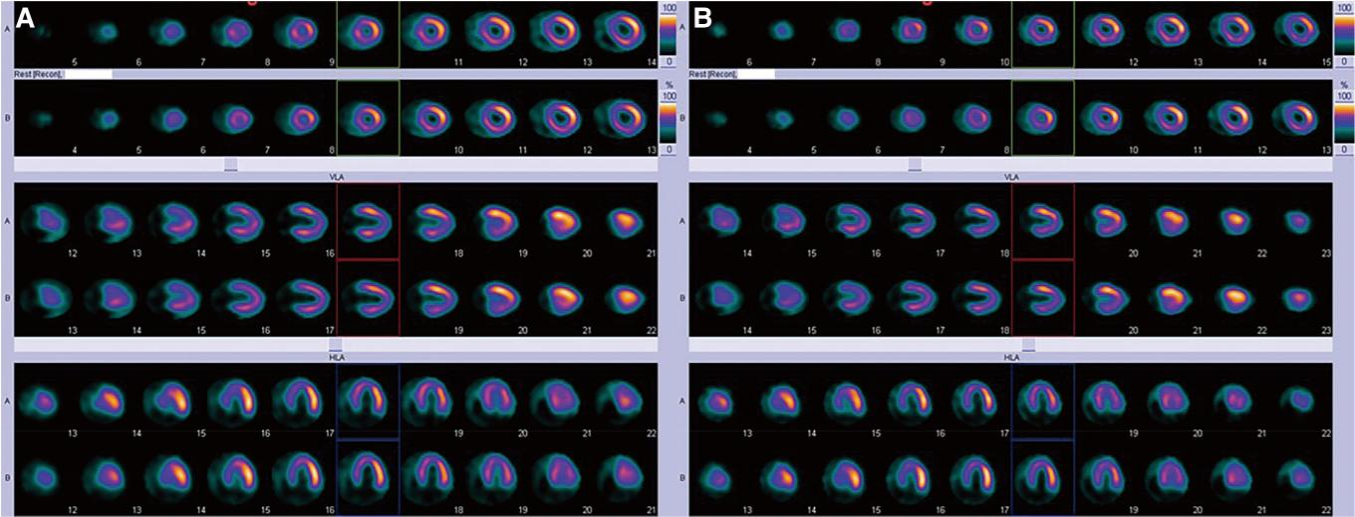
Stress myocardial perfusion scintigraphy before and after transcatheter embolization. Exercise stress myocardial perfusion imaging shows no evidence of inducible ischaemia before (*A*) and after (*B*) fistula embolization. No regional perfusion defects or exercise-induced ischaemic changes were detected in either study.

A 44-year-old woman was referred to our institution for evaluation of exertional dyspnoea and a continuous cardiac murmur. She had noticed shortness of breath over the past 2–3 years, without associated leg oedema, orthopnoea, palpitation, or chest pain. On examination, a continuous murmur was audible from the third left sternal border to the apex, and respiratory sounds were clear. Laboratory testing was within normal limits, including brain natriuretic peptide at 4.8 pg/mL, high-sensitivity troponin T at 0.004 ng/mL, haemoglobin of 14.2 g/dL, and creatinine of 0.62 mg/dL. Electrocardiography revealed sinus tachycardia (heart rate 101 b.p.m.), with increased R + S voltage (3.84 mV) and strain-pattern ST depression in leads V5–6 (see Supplementary material online, Figure  *S1*). Transthoracic echocardiography revealed preserved left ventricular systolic function (ejection fraction 59%) without right heart overload (peak TR velocity 2.35 m/s, estimated right ventricular systolic pressure of 25 mmHg; Supplementary material online, *Video S1*). Contrast-enhanced CT revealed a markedly dilated and tortuous coronary arteriovenous fistula originating from the LCX and draining into the CS (*Figure 1*). A PLSVC was also identified incidentally. Cine cardiac MRI demonstrated a Qp of 5.7 L/min and a Qs of 3.8 L/min, calculating a Qp/Qs ratio of 1.50 (see Supplementary material online, *Table S1*). Stress myocardial perfusion scintigraphy demonstrated no evidence of myocardial ischaemia (*Figure 2A*). Cardiopulmonary testing demonstrated normal cardiopulmonary function, with an oxygen uptake (VO₂) of 10.1 mL/kg/min at the aerobic threshold (heart rate 120 b.p.m., workload 54 W) and a peak VO₂ of 39.0 mL/kg/min (heart rate of 164 b.p.m., workload of 102 W). The maximum heart rate reached 168 b.p.m., with no ischaemic changes or symptoms observed during the test.

Right heart catheterization revealed a cardiac index of 4.6 L/min/m² and Qp/Qs ratio of 1.55, with normal intracardiac pressures, and a decreased systemic vascular resistance index of 1891 dyn·sec·cm⁻⁵·m², consistent with high-output state (haemodynamic pressures and oxygen saturations are summarized in *Table 1*). Coronary angiography confirmed a markedly dilated and tortuous LCX giving rise to left anterior descending and obtuse marginal branches, with the fistulous tract draining into the CS. A small fistulous connection from the right coronary artery to the right atrium was also noted but showed no significant flow or vessel dilation (see Supplementary material online, *Video S2*).

**Table 1:** Haemodynamic pressures and oxygen saturations from right heart catheterization

Measurement site	Pressure (mmHg)	Oxygen saturation (%)
Superior vena cava (a/v/mean)	6/4/4	77.6%
Inferior vena cava (a/v/mean)	6/5/4	84.1%
Right atrium (a/v/mean)	6/4/4	83.4%
Right ventricle (systolic/end-diastolic)	35/5	84.3%
Pulmonary artery (systolic/diastolic/mean)	26/14/20	86.2%
Right pulmonary capillary wedge (a/v/mean)	17/11/12	97.9%
Left ventricle (systolic/end-diastolic)	146/6	N/A
Aorta (systolic/diastolic/mean)	139/84/109	98.7%

Right heart catheterization revealed normal intracardiac pressures. An oxygen saturation step-up was observed from the superior vena cava to the pulmonary artery, indicating a left-to-right shunt. A calculated Qp/Qs ratio of 1.55 with Fick’s principal. An increased cardiac index and decreased systemic vascular resistance index, consistent with high-output physiology.

**Figure 3:**
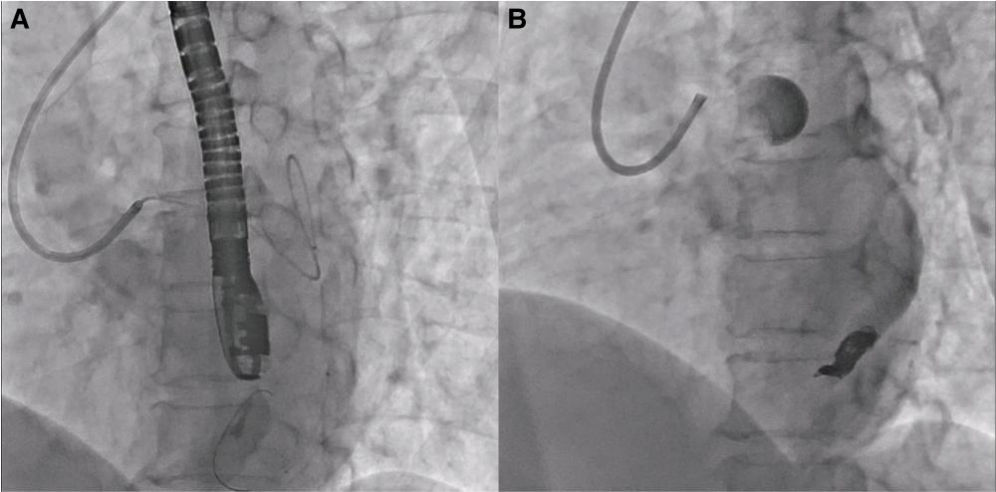
Angiographic images before and after transcatheter coil embolization. A triple coaxial catheter system was established using an ASAHI Hyperion 7Fr SPB3.5 guiding catheter, a TACTICS 3.2/3.4 Fr intermediate catheter, and an Excelsior 1018 microcatheter to achieve stable access to the distal portion of the tortuous fistula and to facilitate precise coil delivery at the targeted neck segment (*A*). Embolization was performed using the following coils: Azur CX Detachable (16 mm × 390 and 8 mm × 280 mm), Target XL Helical Soft (5 mm × 15 cm, two coils), Target Helical ULTRA Coils (2 mm × 8 cm and 2 mm × 4 cm), and an Azur D-18 Helical HydroCoil (3 mm × 5 cm). Post-procedural angiography confirmed complete occlusion of the fistulous connection to the coronary sinus (*B*).

Following a multidisciplinary heart team discussion, closure of the LCX-CS fistula was recommended due to the large shunt volume. After reviewing treatment options including surgical ligation and transcatheter intervention, the patient was scheduled for transcatheter coil embolization. Pre-procedural CT revealed a narrow neck in the distal segment of the fistula, which was selected as the target site for coil deployment. Initially, a venous approach was attempted for flow control. However, catheter navigation from the PLSVC into the CS proved technically challenging, and balloon occlusion failed. A triple coaxial catheter system was employed to facilitate stable navigation and coil delivery via the arterial approach. Embolization was completed, and angiography confirmed complete occlusion of the fistula (*Figure 3*; Supplementary material online, *Video S3*). Warfarin was initiated after the procedure to prevent coronary artery thrombosis.

**Figure 4:**
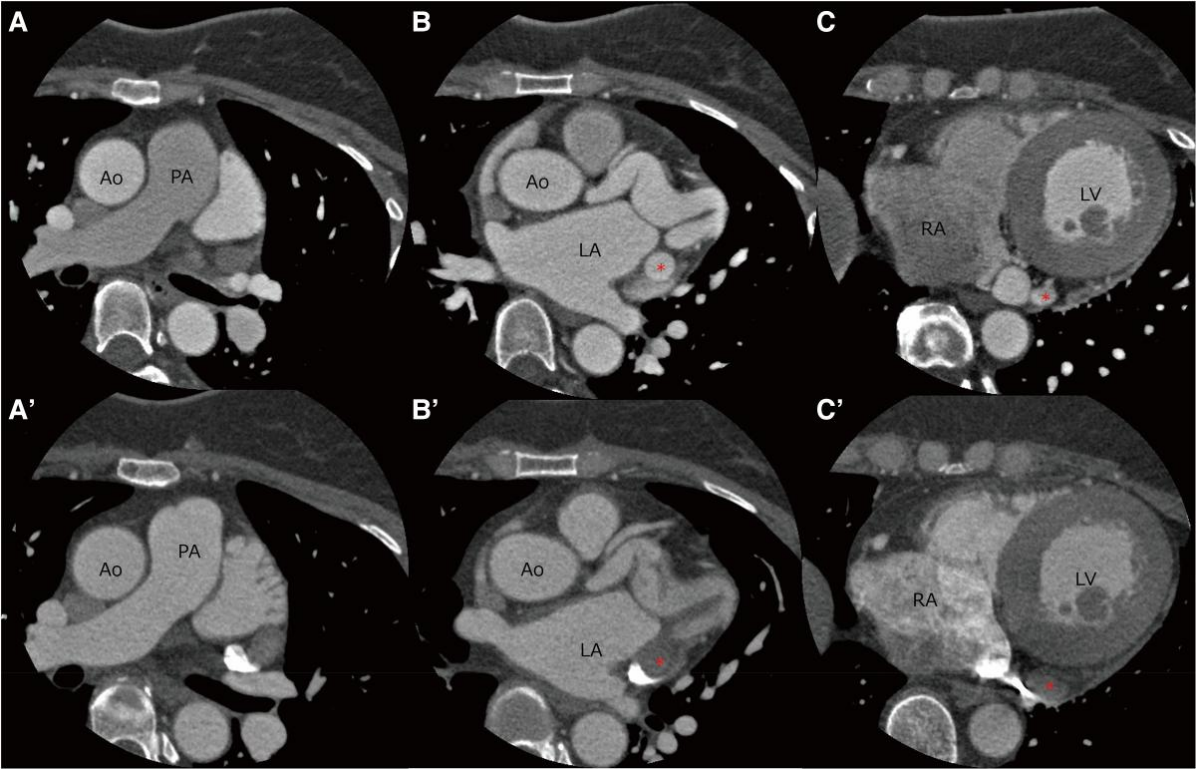
contrast-enhanced computed tomography before and after 6 months of embolization. Contrast-enhanced computed tomography before (*A–C*) and after coil embolization (*A′–C′*). Follow-up contrast-enhanced computed tomography images show the absence of contrast opacification in the previously visualized fistulous vessel (red asterisk), indicating successful occlusion. Ao, aorta; LA, left atrium; LV, left ventricular; PA, pulmonary artery; RA, right atrium.

At 6-month follow-up, cardiac MRI showed a reduced Qp/Qs ratio of 1.17 (Qp = 5.2 L/min, Qs = 4.5 L/min; Supplementary material online, *Table S1*). Contrast-enhanced CT confirmed the absence of contrast opacification in the proximal tract of the coiling target (*Figure 4*), indicating successful fistula closure. Although the patient reported persistent subjective shortness of breath, stress myocardial perfusion scintigraphy remained negative for ischaemia (*Figure 2B*), and cardiopulmonary exercise testing showed no significant change.

## Discussion

Coronary artery fistulas are rare congenital anomalies with an estimated prevalence of 0.1%–0.2% among patients undergoing coronary angiography.^1^ While small CAFs are often asymptomatic, larger fistulas can result in significant left-to-right shunting or myocardial steal, leading to myocardial ischaemia, arrhythmias, or heart failure.^4^ Classically, a Qp/Qs ratio greater than 1.5 has been considered a threshold for surgical closure.^2^ The 2018 AHA/ACC guideline for adult congenital heart disease and the 2020 ESC guidelines for the management of adult congenital heart disease recommend multidisciplinary team discussion about treatment options and considering closure for anatomically large or haemodynamically significant fistulas, even in asymptomatic individuals, to prevent long-term complications.^5,6^ In our case, the patient presented with exertional dyspnoea and had a Qp/Qs ratio of 1.5–1.55 based on both cardiac MRI and right heart catheterization, along with a markedly dilated fistulous vessel. Although myocardial perfusion scintigraphy showed no evidence of ischaemia, the large vessel size and substantial shunt volume supported the decision to proceed with intervention.^3^

Surgical ligation has historically been the standard of care, particularly for large or complex fistulas. However, advances in catheter-based techniques have made transcatheter coil embolization a favourable alternative in appropriately selected cases.^3^ In this case, pre-procedural contrast-enhanced CT provided a detailed understanding of the fistulous anatomy, including its tortuosity and a coiling target of the narrowing neck in its distal segment. This imaging information enabled the selection of a precise embolization target and supported the decision to use a triple coaxial catheter system to ensure stable access and precise coil deployment.^7^ The procedure was successfully completed in a single session without complication. Post-procedural CT confirmed fistula occlusion, and follow-up MRI demonstrated haemodynamic improvement with a Qp/Qs ratio reduced to 1.17.

This case highlights the utility of multimodality imaging for functional and anatomical assessment and procedural planning. Computed tomography allowed detailed visualization of the anatomy, which was critical for selecting the embolization target and planning catheter navigation. Cardiac MRI provided a non-invasive quantitative assessment of left-to-right shunting. Furthermore, both modalities were essential for post-procedural evaluation: CT confirmed anatomical closure, and MRI demonstrated a reduction in shunt flow, supporting the effectiveness of the intervention.

The use of a triple coaxial catheter system enabled stable and precise coil delivery to the distal segment of the tortuous fistula, offering a practical and effective approach for transcatheter closure in selected cases.

## Lead author biography


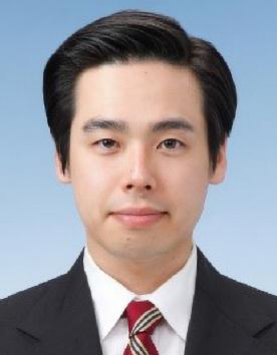


Ryusuke Sekii, MD, graduated from Yokohama City University School of Medicine in 2017. He completed residency training in internal medicine and fellowship training in cardiology in Japan. He is currently a PhD candidate at Yokohama City University, with a research focus on cardiovascular imaging. In 2025, he will begin internal medicine residency training in the USA. His main areas of interests are cardiac imaging and catheter interventions.

## Supplementary Material

ytaf498_Supplementary_Data

## Data Availability

The data underlying this article are available from the corresponding author on reasonable request.
