# Supplementary material for: Multimodality imaging-guided transcatheter closure of a tortuous LCX–CS fistula: a case report
Source: Eur Heart J Case Rep. 2025 Oct 4;9(10):ytaf498. doi: 10.1093/ehjcr/ytaf498 (PMC12532105; doi:10.1093/ehjcr/ytaf498)
Supplement: ytaf498_Supplementary_Data [file ytaf498_supplementary_data.zip › Supplementalmaterial_EHJcase_rerevise.pptx]

## Slide 1
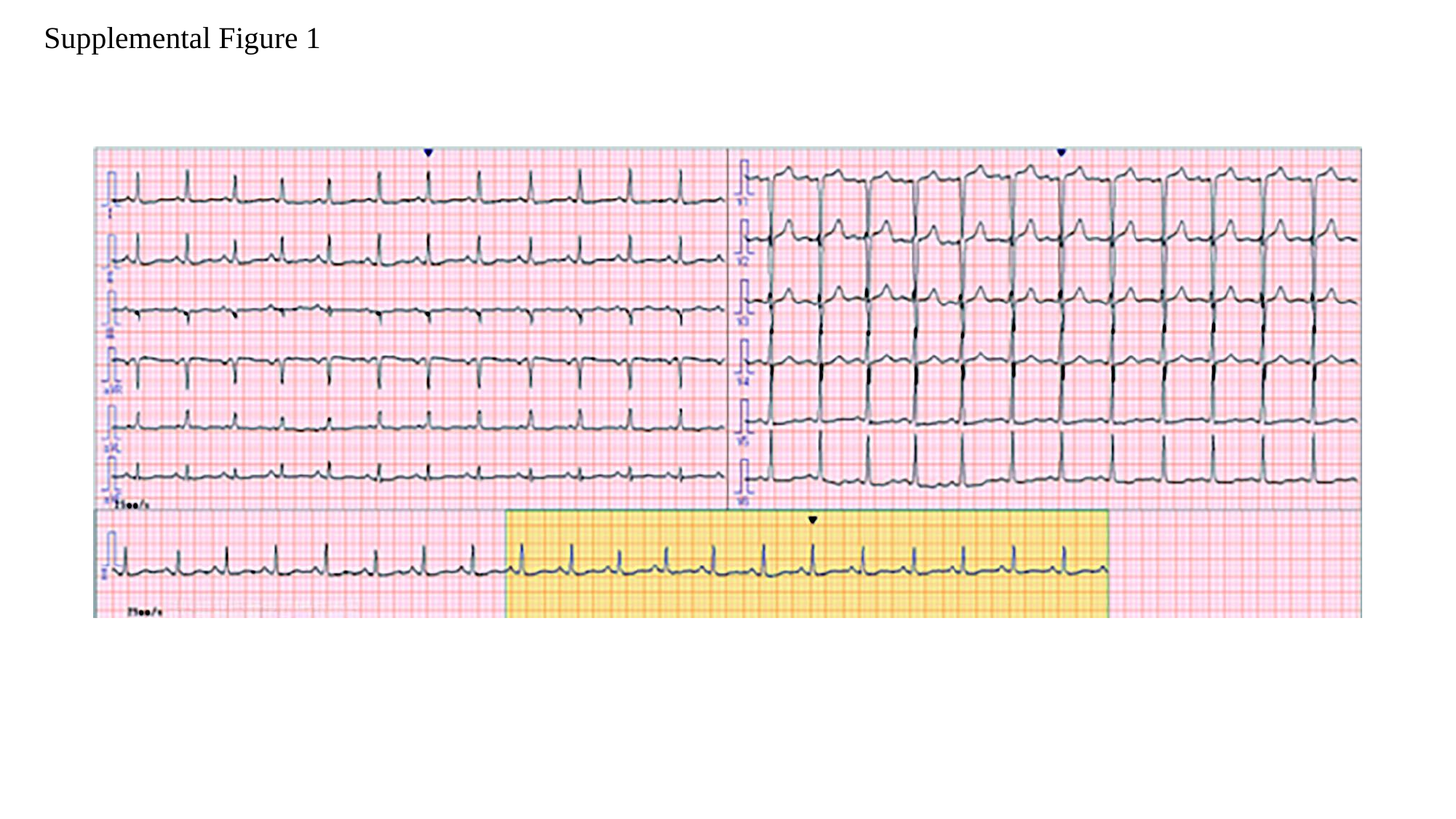

Supplemental Figure 1

## Slide 2
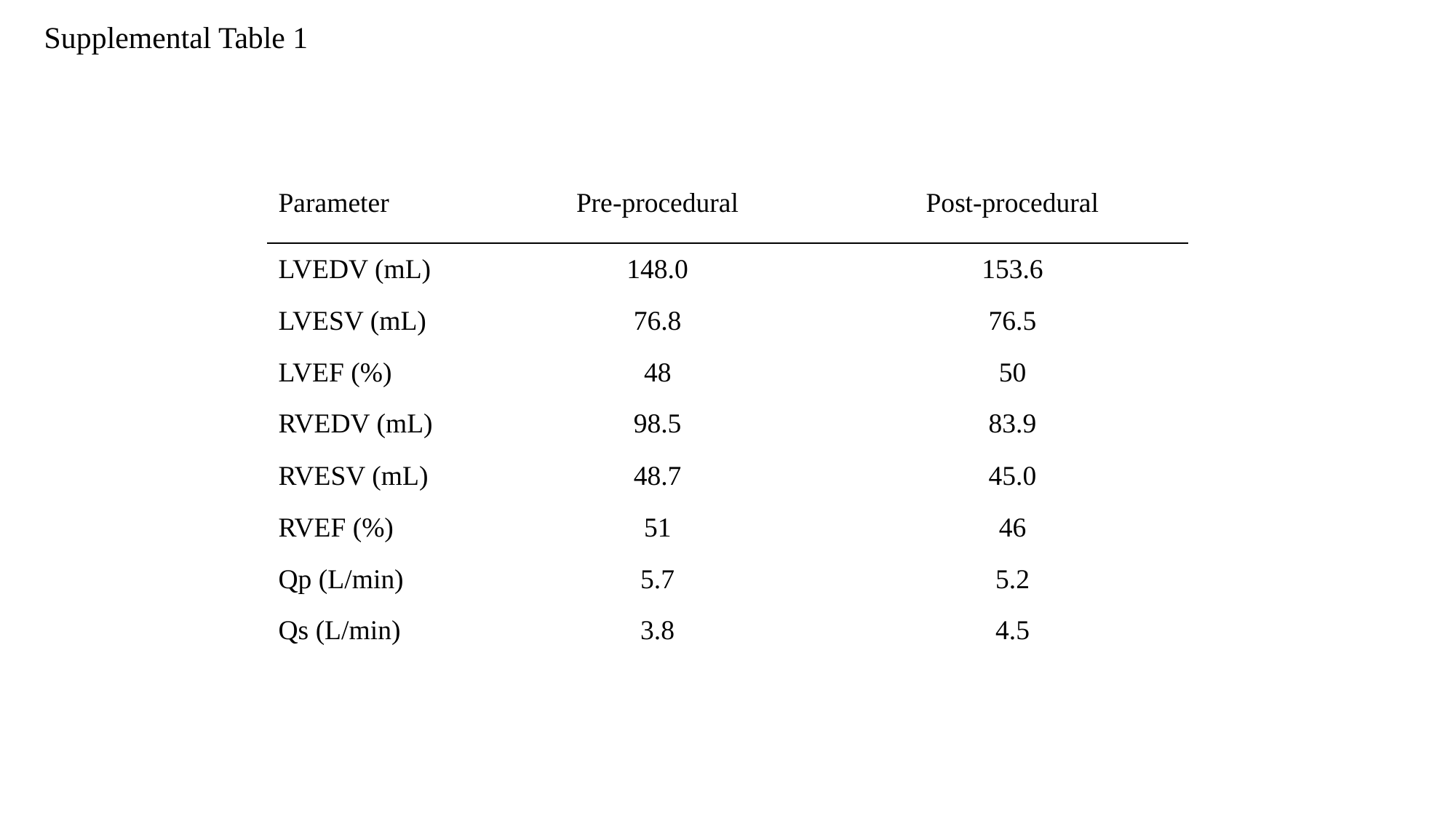

Supplemental Table 1
| Parameter | Pre-procedural | Post-procedural |
| --- | --- | --- |
| LVEDV (mL) | 148.0 | 153.6 |
| LVESV (mL) | 76.8 | 76.5 |
| LVEF (%) | 48 | 50 |
| RVEDV (mL) | 98.5 | 83.9 |
| RVESV (mL) | 48.7 | 45.0 |
| RVEF (%) | 51 | 46 |
| Qp (L/min) | 5.7 | 5.2 |
| Qs (L/min) | 3.8 | 4.5 |
